# Sustained Impairments in Brain Insulin/IGF Signaling in Adolescent Rats Subjected to Binge Alcohol Exposures during Development

**DOI:** 10.4172/2161-0681.1000106

**Published:** 2012-02-10

**Authors:** Alexandra Ewenczyk, Jason Ziplow, Ming Tong, Tran Le, Suzanne M. de la Monte

**Affiliations:** 1 Departments of Pathology (Neuropathology), Neurology, Neurosurgery & Medicine and the Liver Research Center, Rhode Island Hospital and the Warren Alpert Medical School at Brown University, Providence, RI

**Keywords:** Fetal alcohol syndrome, Adolescence, Brain development, Motor function, Insulin signaling, Central nervous system, Receptor binding, Brain insulin resistance, Cerebellum

## Abstract

**Background:**

Chronic or binge ethanol exposures during development can cause fetal alcohol spectrum disorder (FASD) which consists of an array of neurobehavioral deficits, together with structural, molecular, biochemical, and neurotransmitter abnormalities in the brain. Previous studies showed that perinatal neurodevelopmental defects in FASD are associated with inhibition of brain insulin and insulin-like growth factor (IGF) signaling. However, it is not known whether sustained abnormalities in adolescent brain structure and function are mediated by the same phenomena.

**Aims:**

Using an early postnatal (3^rd^ trimester equivalent) binge ethanol exposure model, we assessed neurobehavioral function, structure, and the integrity of insulin/IGF signaling in young adolescent cerebella.

**Methods:**

Long Evans male rats were treated with 50 µl of saline (vehicle) or 2 mg/kg of ethanol by i.p. injection on postnatal days (P) 2, 4, 6, and 8. On P19–20, rats were subjected to rotarod testing of motor function, and on P30, they were sacrificed to harvest cerebella for histological, molecular, and biochemical studies.

**Results:**

Binge ethanol exposures impaired motor function, caused sustained cerebellar hypocellularity, and reduced neuronal and oligodendrocyte gene expression. These effects were associated with significant deficits in insulin and IGF signaling, including impaired receptor binding, reduced Akt, and increased GSK-3β activation.

**Conclusions:**

FASD-associated neurobehavioral, structural, and functional abnormalities in young adolescent brains may be mediated by sustained inhibition of insulin/IGF-1 signaling needed for cell survival, neuronal plasticity, and myelin maintenance.

## Introduction

Alcohol misuse during pregnancy causes significant neurodevelopmental abnormalities including microcephaly, cerebellar hypoplasia, motor deficits, and neuro-cognitive impairments ranging from attention deficit hyperactivity disorder to mental retardation. This pathology, combined with various stereotypical craniofacial defects is termed, ‘fetal alcohol spectrum disorders’ (FASD) [1,2]. Long-term consequences of ethanol’s selective targeting of the temporal lobe, hippocampus, and cerebellum include sustained deficits in cognitive and motor function [3] that lead to behavioral problems, poor achievement, and problematic social and academic outcomes in children, adolescents, and young adults [4–6].

One of the key adverse effects of ethanol on the immature central nervous system (CNS) is to profoundly inhibit insulin and insulin-like growth factor (IGF) signaling pathways [7]. Insulin and IGF regulate a broad array of cellular functions in the immature brain, including neuronal survival and differentiation, myelin formation and maintenance, neuronal migration, plasticity, metabolism, mitochondrial function, and neurotransmitter homeostasis and responsiveness [8–16]. Previous studies showed that ethanol inhibits insulin and IGF signaling at multiple points within the cascade, beginning at the receptor level and extending downstream through pathways that regulate growth, survival, energy metabolism, neuronal migration, and plasticity [17–22]. More specifically, ethanol mediates its adverse effects on insulin and IGF-1 signaling by: 1) inhibiting phosphorylation and activation of corresponding receptor tyrosine kinases (RTKs), and their immediate down-stream effector molecules, including insulin receptor substrate (IRS) proteins [23,24]; 2) inhibiting signaling through IRS-associated phosphotidyl-inositol-3-kinase (PI3K) with attendant reduced activation of Akt and increased activation of glycogen synthase kinase 3β (GSK-3β) [7,19,23–28]; and 3) increasing activation of phosphatases that negatively regulate RTKs (PTP-1b) and PI3K (PTEN) [24–26]. Akt promotes cell survival, cell migration, energy metabolism, and neuronal plasticity, and it inhibits GSK-3β activity, which when aberrantly increased causes oxidative stress and apoptosis [16]. In essence, ethanol’s inhibitory effect on insulin and IGF-1 receptor signaling produces a state of insulin/IGF resistance, and thereby accounts for several major CNS abnormalities in FASD [2,18,29–33].

Previous studies focused on the effects of chronic prenatal ethanol exposure in relation to cerebellar structure and gene expression in the perinatal period, shortly after birth [24,30,34]. However, it has been well documented that either chronic or binge ethanol exposures during development can have significant long-term adverse consequences with respect to neurobehavioral function in adolescents [2,29], yet the mediators of such responses are poorly understood. Since chronic ethanol exposures in adult humans and experimental animals also cause brain insulin/IGF resistance with reduced signaling downstream through IRS-PI3K-Akt, neuronal loss, impaired mitochondrial and neurotransmitter functions, and increased oxidative stress [31,35], we hypothesized that similar abnormalities might persist in young adolescent brains, even in the absence of subsequent developmental exposures to ethanol. Herein, using a binge ethanol exposure model in which rat pups were exposed to ethanol in the early postnatal period, we assessed the potential role of persistent insulin/IGF resistance as a mediator of impaired cerebellar motor function in the early adolescent period.

## Materials and Methods

### Materials

Qiazol reagent, EZ1 RNA universal tissue kit, QuantiTect SYBR Green polymerase chain reaction (PCR) master mix, and the BIO Robot Z1 were from Qiagen Inc (Valencia, CA). Histofix was purchased from Histochoice (Amresco, Solon, OH). The AMV first strand cDNA synthesis kit was obtained from Roche Diagnostics Corporation (Indianapolis, IN). The Akt Pathway Total and Phospho 7-Plex panels were purchased from Invitrogen (Carlsbad, CA). Bicinchoninic acid (BCA) reagents were from Pierce Chemical Corp. (Rockford, IL). All other fine chemicals were purchased from CalBiochem (Carlsbad, CA), Pierce (Rockford, IL), or Sigma (St. Louis, MO).

### Early postnatal binge ethanol exposure model

Long Evans rats were used to generate a human 3^rd^ trimester-equivalent binge ethanol exposure model. At birth, litters were culled to 8 pups per dam. Pups from 12 different litters were administered intra-peritoneal (i.p.) injections (50 µl) of sterile saline or 2 mg/kg ethanol (diluted in saline) on postnatal days (P) 2, 4, 6, and 8 [36–38]. Injections were made at the same time each day between 12 PM and 2 PM to control for diurnal fluctuations in stress responses. Rats were weighed twice weekly. After weaning, rats were pair-fed with regular chow.

### Motor function assessment

On P19, rats were trained to remain balanced on the rotating Rotamex-5 apparatus (Columbus Instruments) at 1–5 rpm. On P20, rats (N=8–10 per group) were administered 10 trials at incremental speeds up to 10 rpm, with 10 minutes rest between each trial. The latency to fall was automatically detected with photocells placed over the rod. However, trials were stopped after 30 seconds to avoid exercise fatigue. Data from trials 1–3 (2–5 rpm), 4–7 (5–7 rpm), and 8–10 (8–10 rpm) were culled and analyzed using the Mann-Whitney test. On P30, rats were sacrificed by isofluorane inhalation, and cerebella were harvested for histological, biochemical and molecular studies. Freshly isolated cerebella were hemisected in the mid-sagittal plane; one hemisphere was snap frozen in a dry ice-methanol bath and stored at −80°C for later RNA and protein studies, and the other half was immersion fixed in Histofix. The Lifespan-Rhode Island Hospital IACUC committee approved these procedures and the use of rats in experiments.

### Quantitative reverse transcriptase polymerase chain reaction (qRT-PCR) assays

We used qRT-PCR analysis to measure relative mRNA abundance corresponding to Hu (neuronal gene), myelin-associated glycoprotein 1 (MAG-1) for oligodendrocytes, glial fibrillary acidic protein (GFAP) for astrocytes, allograft inflammatory factor 1 (AIF1) for activated microglia, acetyl cholinesterase (AChE), choline acetyltransferase (ChAT), insulin, IGF-1, and IGF-2 polypeptides and receptors, and insulin receptor substrates (IRS), types 1, 2, and 4 [39–41]. Gene specific primer pairs were designed using MacVector 10 software (MacVector, Inc., Cary, NC) and target specificity was verified using NCBI-BLAST (Basic Local Alignment Search Tool). The amplified signals were detected and analyzed using the Master cycler ep realplex instrument and software (Eppendorf AG, Hamburg, Germany). Relative mRNA abundance was calculated from the ng ratios of specific mRNA to 18S rRNA measured in the same samples. Assays were performed in triplicate.

### Multiplex ELISA

We used bead-based multiplex ELISAs to examine the integrity of insulin and IGF-1 signaling networks by measuring immunoreactivity to the insulin receptor (IR), IGF-1 receptor (IGF-1R), IRS-1, Akt, glycogen synthase kinase 3β (GSK-3β), ^pYpY1162/1163^-IR, ^pYpY1135/1136^-IGF-1R, ^pS312^-IRS-1, ^pS473^-Akt, and ^pS9^-GSK-3β according to the manufacturer’s protocol. Brain tissue samples were homogenized in lysis buffer (50 mM Tris-HCl, pH 7.5, 1% Triton X-100, 2 mM EGTA, 10 mM EDTA, 100 mM NaF, 1 mM Na_4_P_2_O_7_, 2 mM Na_3_VO_4_) containing protease and phosphatase inhibitors [42]. 200 µg proteins in 100 µl of lysis buffer were incubated with antibody-bound beads. Captured antigens were detected with biotinylated secondary antibody and phycoerythrinconjugated Streptavidin. Plates were read in a Bio-Plex 200 system (Bio-Rad, Hercules, CA). Data are expressed as fluorescence light units (FLU) corrected for protein concentration.

### Receptor binding assays

Insulin, IGF-1, and IGF-2 receptor binding in the brain was measured using competitive saturation assays [41]. Membrane proteins extracted from fresh frozen tissue were incubated in 100 µl reactions containing binding buffer and 0.0031 to 1 µCi/ml of ^125^I (2000 Ci/mmol) insulin, IGF-1, or IGF-2. Non-specific binding was measured in duplicate reactions containing excess (0.1 µM) cold ligand. Radioactivity was measured in polyethylene glycol 8000 precipitates (bound ligand) and the corresponding supernatants (free ligand) in an LKB CompuGamma CS Gamma counter. Specific binding was calculated by subtracting fmols bound in the presence of excess cold ligand (non-specific), from fmols bound in the absence of cold ligand (total). Best-fit analysis predicted a one-site model, and Scatchard analysis was used to calculate saturation binding (BMAX) and binding affinity (Kd). Binding assay results were analyzed using the Graph Pad Prism 5 software (Graph Pad Software, Inc., San Diego, CA).

### Statistics

The experimental model was generated with 12 litters (8 pups each) in which 2 each were treated with vehicle or ethanol. All assays were performed with 12 samples per group. Data corresponding to levels of gene expression or immunoreactivity are depicted in boxplot graphs representing the medians (horizontal bars), 95% confidence intervals (box limits), and range (whiskers) for each group. Inter-group comparisons were made using Student t-test. Statistical analyses were performed using the Graph Pad Prism 5 software (San Diego, CA) and significant P-values (<0.05) are indicated within the graph panels.

## Results

### Early postnatal binge ethanol exposures lead to impaired motor function in young adolescent rats

**Figure 1:**
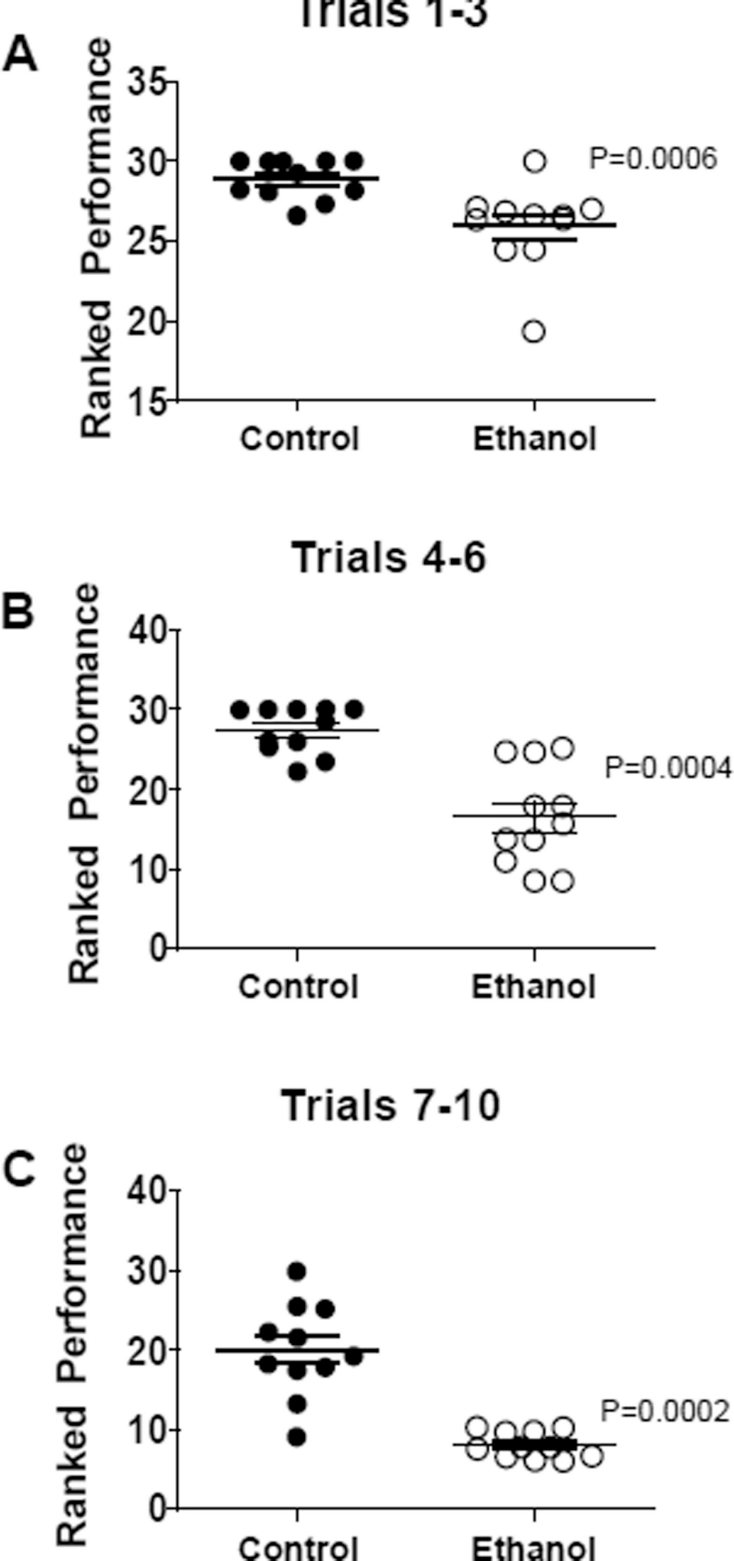
Long-term effects of early postnatal binge ethanol exposure on rotarod motor performance. Long Evans rat pups were treated with 50 µl i.p. injections of saline (vehicle) or 2.0 mg/kg ethanol in saline on postnatal days 2, 4, 6, and 8. On postnatal 19, rats (N=12/group) were subjected to 10 rotarod test trials in which speed of the rotating rod was incremented with each trial. Latency to fall was recorded. Data from Trials 1–3 (2–5 rpm), Trials 4–6 (5–7 rpm), or Trials 7–10 (8–10 rpm) were culled and analyzed using the Mann-Whitney test. Significant inter-group differences are shown in the panels.

Data from rotarod tests of cerebellar function were grouped into trials according to the speed of the rotating bar. For all 3 trial clusters, the ethanol-exposed group exhibited significantly reduced latencies to fall relative to control (Figure 1). In Trials 1–3, which were the least challenging, the differences between the control and ethanol-exposed group were modest but statistically significant (Figure 1A). For Trials 4–6, performance among controls was similar to that observed for Trials 1–3, whereas for the ethanol-exposed group, mean latency to fall was further reduced (Figure 1B). Finally, for Trials 7–10, which were the most challenging, although performance among controls declined relative to earlier trials, the ethanol-exposed group exhibited its worst performance, and the shortest mean latency to fall (Figure 1C).

### Effects of early postnatal binge ethanol exposures on cerebellar structure in young adolescent rats

**Figure 2:**
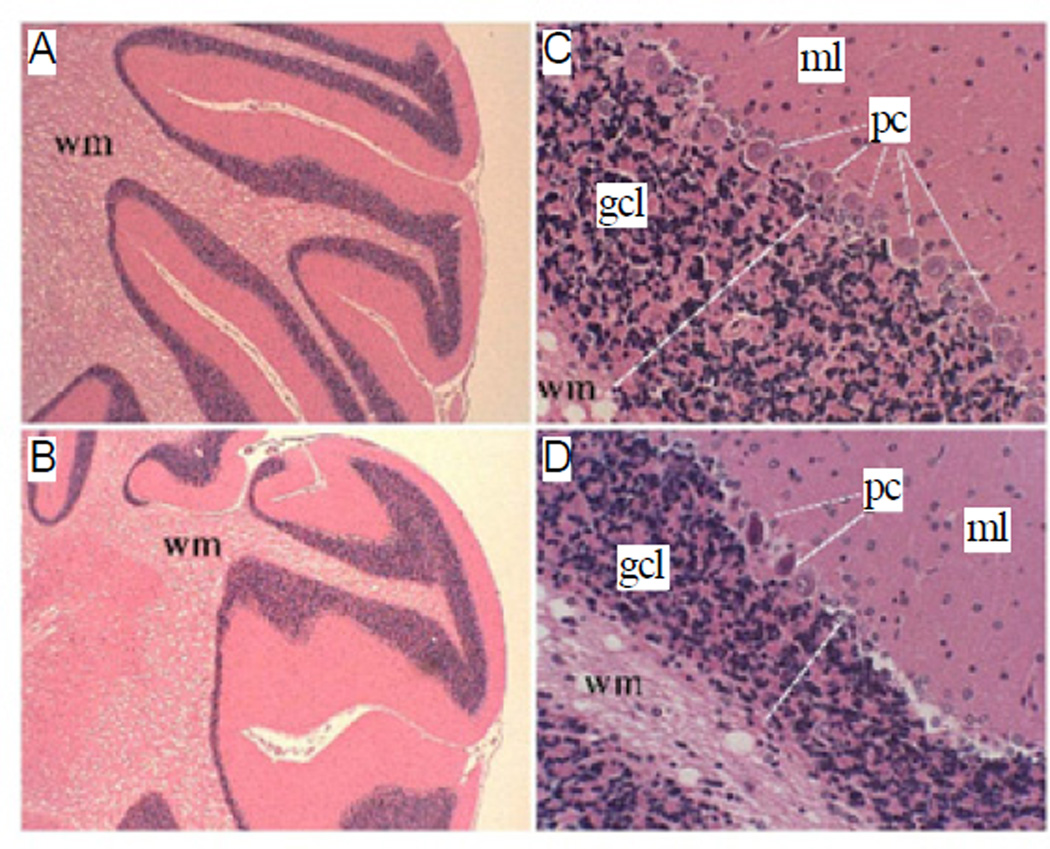
Sustained structural abnormalities in adolescent cerebella following early postnatal binge ethanol exposures. Long Evans rat pups were treated with 50 µl i.p. injections of saline (vehicle) or 2.0 mg/kg ethanol in saline on postnatal days 2, 4, 6, and 8. Rats were sacrificed on P30 and cerebella were fixed and embedded in paraffin. Histological sections were stained with Hematoxylin and Eosin. (A, B) Low (original, 40×) and (C, D) high (original, 160×) magnification images demonstrating (A, B) control cerebella with long slender folia with uniform thickness of the molecular layer (ml), and well-populated Purkinje (pc) and granule cell (gc) layers in cortex, compared with (C, D) ethanol-exposed cerebella, which had shallow, blunted and simplified folia with irregular thickness of the molecular layer, irregular thinning of the granule cell layer (gcl) and white matter (wm) cores, and numerous gaps corresponding with loss of neurons in the Purkinje cell layer. White lines span the thicknesses of the granule cell layers.

Histological sections of P30 rat cerebella demonstrated slender folia with deep and complex sulci (grooves), uniform molecular layers and white matter cores, and well-populated Purkinje and granule cell layers in the cortex (Figures 2A, 2C). In contrast, cerebella of ethanol-exposed rats exhibited conspicuous blunting and simplification of the folia with irregular white matter cores and thicknesses of the molecular and granule cell layers, and numerous gaps corresponding to neuronal loss in the Purkinje cell layer (Figures 2B, 2D). In addition, many residual Purkinje cells had eosinophilic cytoplasms with pyknotic nuclei, corresponding to changes associated with early necrosis.

### Reduced neuronal and oligodendrocyte gene expression following early postnatal binge ethanol exposures

**Figure 3:**
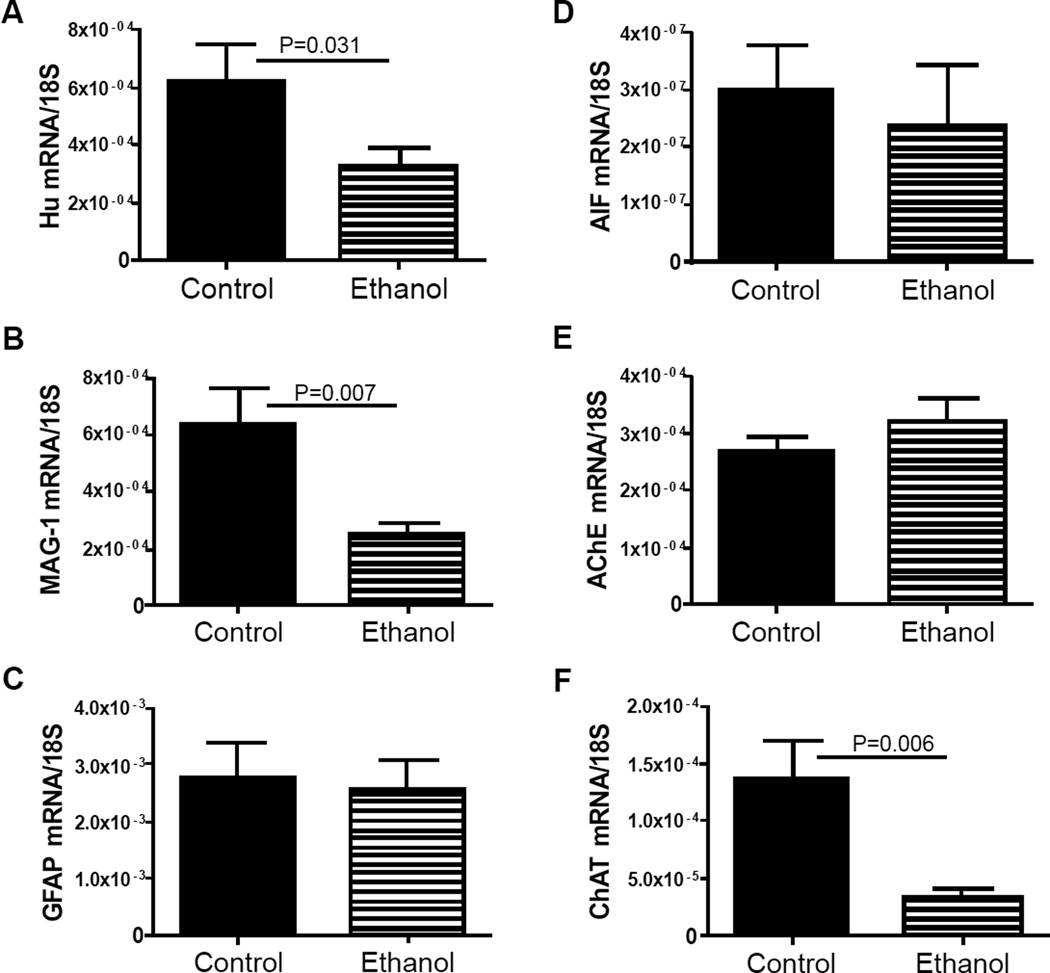
Early postnatal binge ethanol exposures alter neuronal and glial gene expressions in early adolescent cerebella. RNA extracted from cerebella (N=8 samples per group) was reverse transcribed, and the cDNAs were used to measure gene expression by qPCR analysis. Results were normalized to 18S rRNA measured in parallel reactions. Graphs depict relative levels of gene expression for (A) neuronal Hu, (B) myelin-associated glycoprotein-1 (MAG-1), (C) glial fibrillary acidic protein (GFAP), (D) allograft inflammatory factor -1 (AIF), (E) acetylcholinesterase (AChE), and (F) choline acetyltransferase (ChAT), and. Inter-group comparisons were made using Student t-tests. Significant P-values are shown over the graphs.

mRNA transcripts corresponding to Hu (neurons), myelin-associated glycoprotein 1 (MAG-1; oligodendrocytes), glial fibrillary acidic protein (GFAP; astrocytes), allograft inflammatory factor 1 (AIF-1; activated microglia), acetyl cholinesterase (AChE), and choline acetyltransferase (ChAT) were measured by qRT-PCR analysis with results normalized to 18S rRNA (Figure 3). These genes were selected for study to help gauge the long-term impact of developmental exposure to ethanol on cerebellar structure, particularly with regard to survival of neurons and oligodendrocytes. Cholinergic neurotransmitter function was also assessed because acetylcholine is a major neurotransmitter utilized for cerebellar motor functions [43,44]. Corresponding with the ethanol-associated motor impairments, cerebellar atrophy, cell loss in Purkinje and granule cell cortical layers, and irregular structure of the subcortical white matter, Hu, MAG-1, and ChAT mRNA levels were significantly reduced in the ethanol-exposed cerebella (Figures 3A, 3B, 3F). In contrast, GFAP (Figure 3C), AIF-1 (Figure 3D), and AChE (Figure 3E) mRNA levels were similar in control and ethanol-exposed cerebella. These findings suggest that early postnatal binge ethanol exposure-induced impairments in young adolescent motor function were mediated in part by reduced populations of cholinergic neurons rather than increased degradation of acetylcholine.

### Early postnatal binge ethanol exposure impairs insulin/IGF signaling in young adolescent cerebella

**Figure 4:**
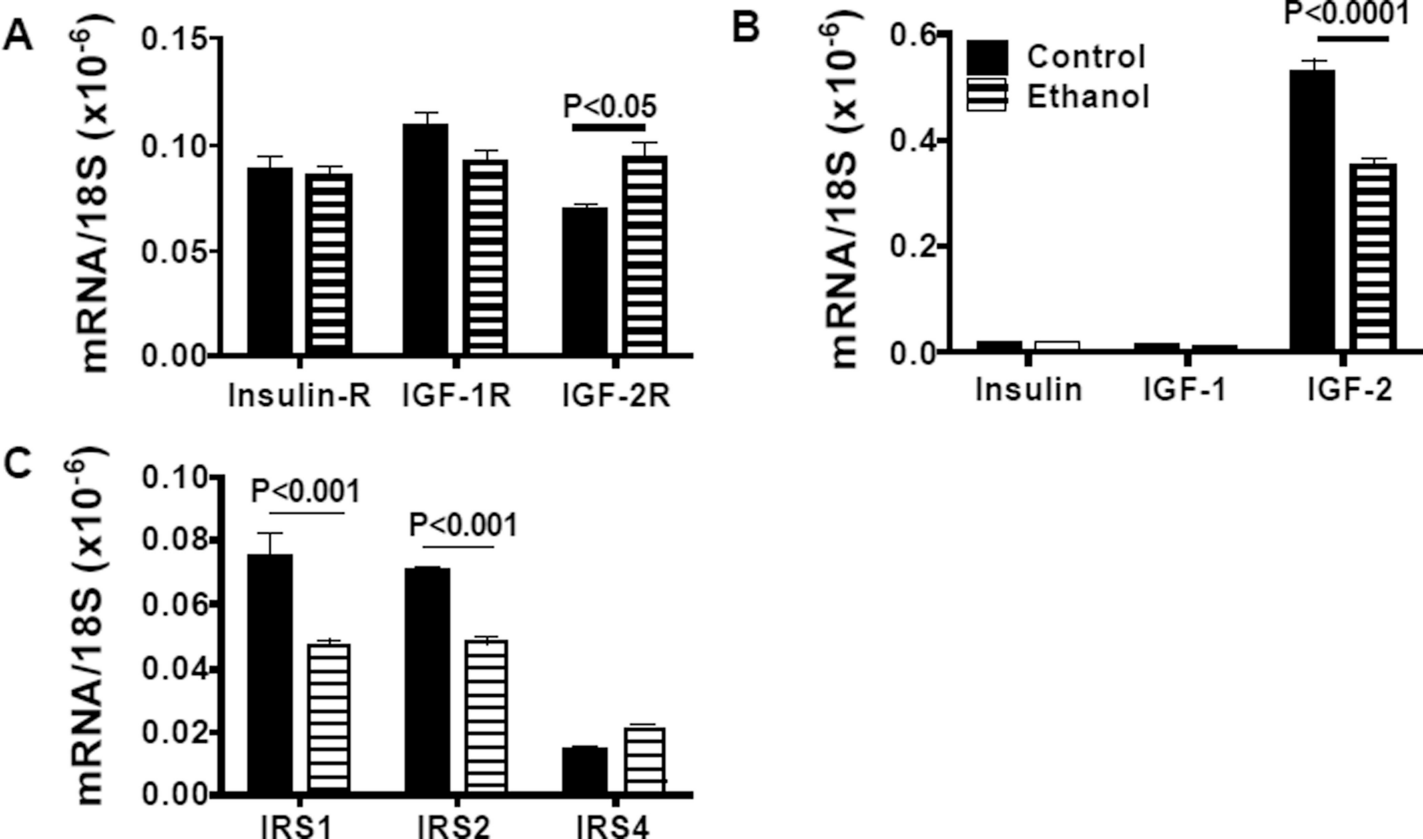
Effects of early postnatal binge ethanol exposures on expression of insulin, insulin-like growth factor-1 (IGF-1), IRS-2, and insulin receptor substrate genes in young adolescent cerebella. RNA extracted from cerebella (N=8 samples per group) was reverse transcribed, and the cDNAs were used to measure gene expression corresponding to the (A) insulin, IGF-1, and IGF-2 polypeptides, (B) insulin, IGF-1, and IGF-2 receptors, and (C) IRS1, IRS2, and IRS4. Results were normalized to 18S rRNA measured in parallel reactions. Graphs depict relative levels of gene expression Inter-group comparisons with respect to trophic factors, receptors, or IRS molecules were made by repeated measures two-way ANOVA tests with the Bonferroni post hoc significance test. Significant P-values are shown over the graphs.

We used qRT-PCR analysis to measure expression of insulin and IGF trophic factors and receptors, and IRS molecules as previously described [24,28,35]. Due to their inter-relatedness, data corresponding to the trophic factors, receptors, or IRS genes were grouped and analyzed by two-way ANOVA with the Bonferroni post-hoc significance test (Figure 4). Insulin and IGF-1 receptors were similarly expressed in control and ethanol-exposed cerebella, whereas IGF-2R mRNA transcripts were significantly more abundant in ethanol-exposed relative to control cerebella (Figure 4A). In both control and ethanol-treated rats, insulin and IGF-1 polypeptide mRNA transcripts were similarly low-level in abundance compared with IGF-2 (Figure 4B). The mean level of IGF-2 mRNA was significantly lower in the ethanol-exposed relative to control cerebella. In ethanol-exposed cerebella, both IRS1 and IRS2 mRNA levels were significantly reduced relative to control, while IRS4 expression was similar in the two groups (Figure 4C).

**Figure 5:**
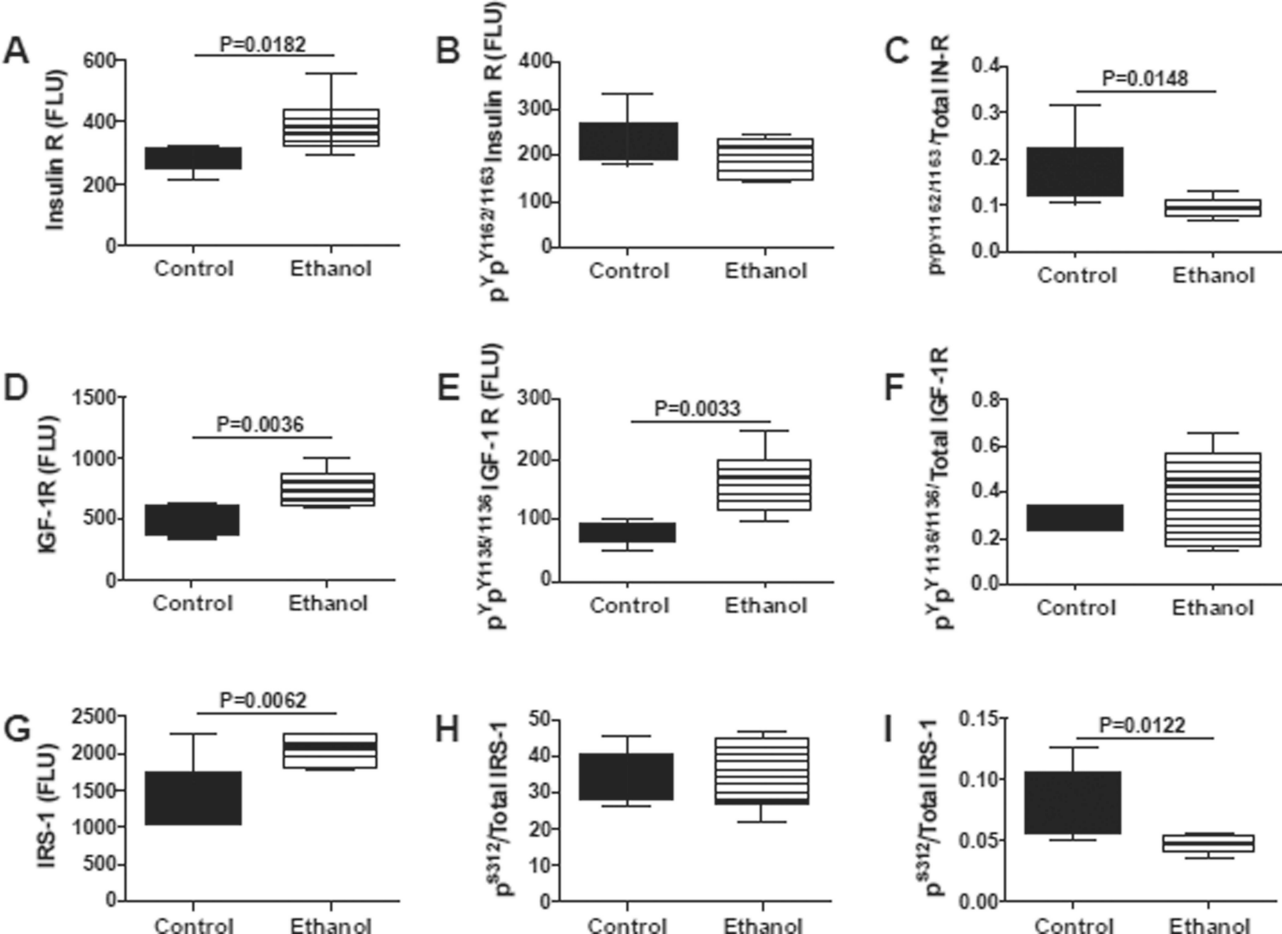
Sustained insulin and IGF-1 resistance in young adolescent cerebella following early postnatal binge ethanol exposures Cerebella protein homogenates were used to measure immunoreactivity to (A) insulin receptor (IR), (D) IGF-1R, (G) IRS-1, (B) pYpY1162/1163-IR, (E) pYpY1135/1136-IGF-1R, (H) pS312-IRS-1 using the bead-based Akt and phospho-specific Akt pathway multiplex ELISA kits. Phospho-/total protein ratios for (C) IR, (F) IGF-1R, and (I) IRS-1 were calculated. Comparisons (N=8 samples per group) were made using Student T-tests. Significant differences are indicated within the panels.

**Figure 6:**
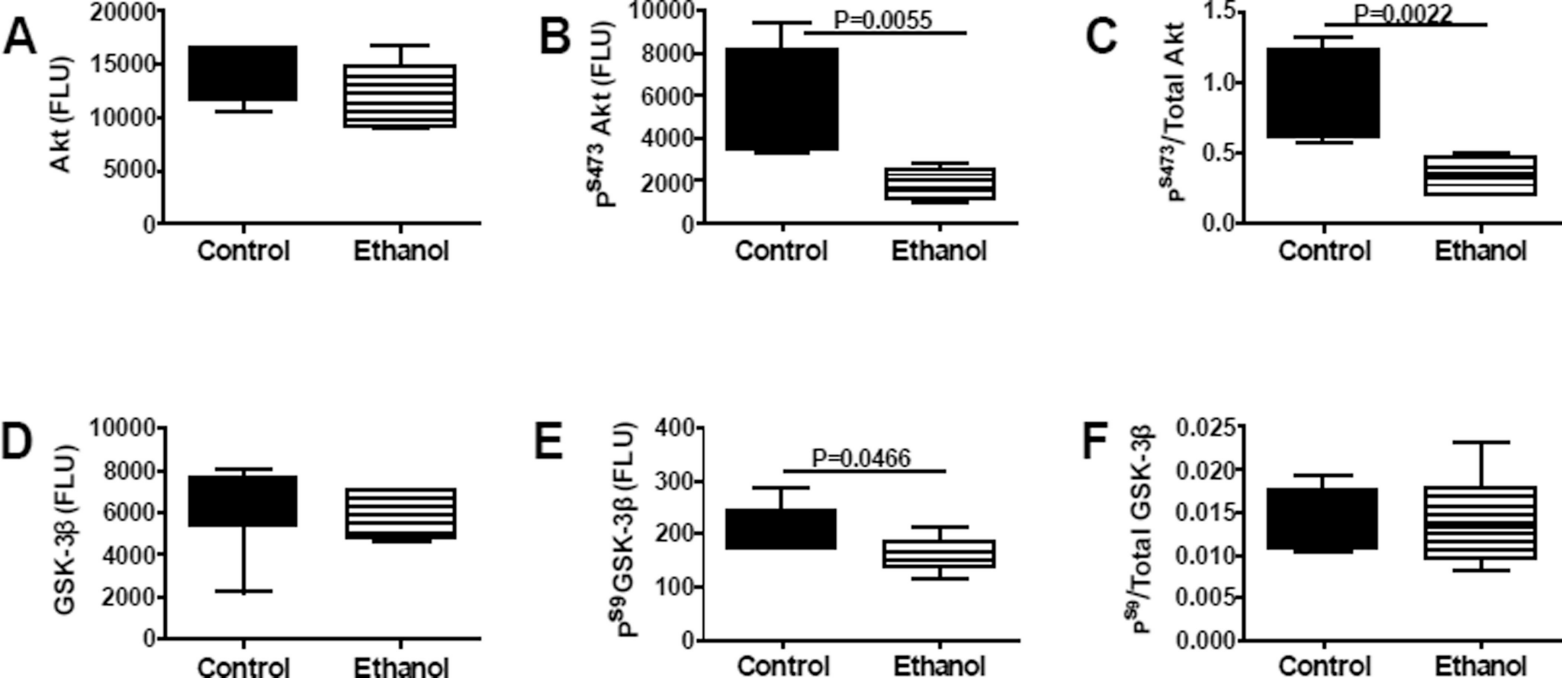
Effects of early postnatal binge ethanol exposures on signaling through Akt and GSK-3β in young adolescent cerebella. Cerebellar protein homogenates were used to measure immunoreactivity to (A) Akt, (B) pS473-Akt, (D) GSK-3β, and (E) pS9-GSK-3β using bead based total Akt and phospho-specific Akt multiplex ELISA kits. In addition, relative degrees of phosphorylation represented by (C) pS473/Total Akt and (F) pS9/Total GSK-3β were calculated. Inter-group comparisons were made using Student T-tests. Significant differences are indicated within the panels.

We used multiplex ELISAs to further interrogate ethanol’s long-term effects on the integrity of insulin/IGF signaling in the cerebellum. We measured total and phosphorylated levels of insulin receptor (pY1162/pY1163), IGF-1 receptor (pY1135/pY1136), IRS-1 (pS312), Akt (pS473), and GSK-3β (pS9), and calculated the relative levels of phosphorylation from the ratios of phospho-/total protein (Figures 5 and 6). Early postnatal binge ethanol exposures significantly increased cerebellar insulin receptor (Figure 5A), IGF-1 receptor, IRS-1 (Figure 5C), and pYpY1135/1136-IGF-1R (Figure 5E), and reduced the relative levels (ratios) of pYpY1162/1163 to total insulin receptor (Figure 5G) and pS312/total IRS-1 (Figure 5I) relative to control. In contrast, no significant inter-group differences were observed with respect to pYpY1162/1163 insulin receptor (Figure 5D) and pS312 IRS-1 (Figure 5F).

Insulin, IGF-1 and IRS-1 signal downstream to activate Akt and inhibit GSK-3β through phosphorylation of specific Ser residues on these proteins. In addition, signaling through Akt and GSK-3β can be regulated by altering the expression levels of these proteins. Multiplex ELISAs demonstrated that early postnatal binge ethanol exposures significantly reduced cerebellar levels of pS473-Akt (Figure 6C), pS473/ total Akt (Figure 6E), and pS9-GSK-3β (Figure 6D). In contrast, the mean levels of total Akt (Figure 6A), GSK-3β (Figure 6B), and pSer9/ total GSK-3β (Figure 6F) were similar in control and ethanol-exposed cerebella.

**Figure 7:**
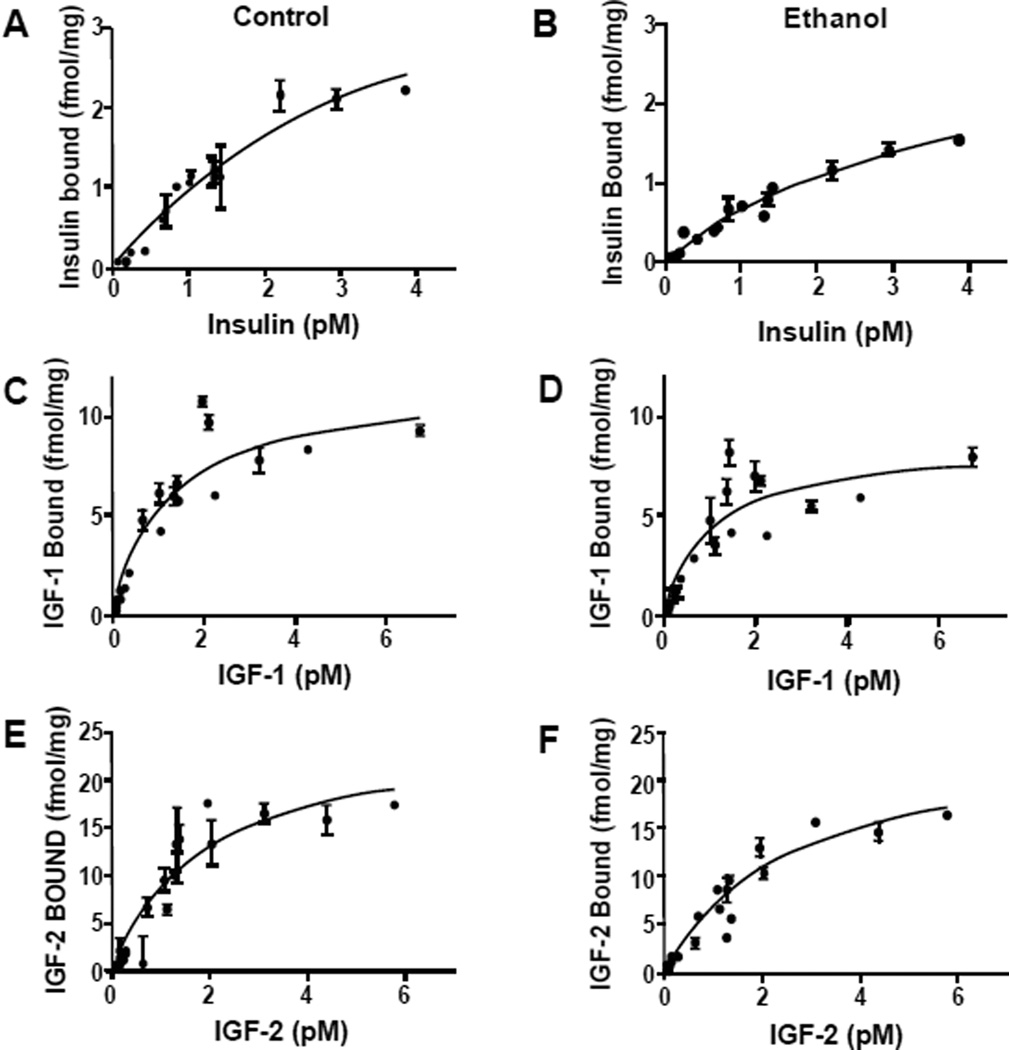
Impaired insulin and IGF-1 receptor binding in young adolescent cerebella following early postnatal binge ethanol exposures Competitive saturation binding assays were performed by incubating cerebellar membrane protein extracts with 0.5–500 pM [125I]-labeled insulin, IGF-1, or IGF-2 as tracer, in the presence or absence of 100 nM cold ligand. Membrane bound tracer was precipitated and radioactivity in the supernatants (containing free ligand) and pellets (containing bound ligand) was measured in a gamma counter. Graphs depict specific binding (fmol/mg protein) ± S.D. relative to pM input of radiolabeled (A–B) insulin, (C,D) IGF-1, and (E,F) IGF-2 in (A,C,E) control (vehicle-treated) and (B,D,F) ethanol-exposed samples. The calculated binding indices and inter-group statistical comparisons are provided in Table 1.

Competitive saturation binding assays were used to examine long-term effects on insulin, IGF-1, and IGF-2 receptor binding in the cerebellum, as previous studies linked impairments in receptor binding to ethanol-induced insulin and IGF resistance in liver and brain [28,35,45]. Non-linear curve fitting analysis predicted single-site specific binding for each receptor in both groups; the goodness of fits (R^2^) ranged from 0.82 to 0.94 (Table 1). Scatchard analysis was used to calculate Bmax (top-level binding) and Kd (binding affinity). In control and ethanol-exposed cerebella, the highest top-level (Bmax) binding was observed for IGF-2R, followed by IGF-1R, and then insulin receptor (Figure 7 and Table 1). Early postnatal binge ethanol exposures significantly reduced the Bmax for insulin (P<0.05) and IGF-1 (P<0.01), whereas the Bmax for IGF-2 receptor binding was nearly the same for control and ethanol exposed cerebella. In contrast, there were no significant inter-group differences found with respect to receptor binding affinity (Kd) (Table 1).

## Discussion

### Effects of early postnatal binge ethanol exposures on adolescent cerebellar function and structure

This study demonstrates that early postnatal binge ethanol exposures cause sustained structural and functional abnormalities in the cerebellum. The Structural damage was characterized by blunting and simplification of cerebellar folia with reduced thickness of the granule cell layer and reduced neuronal densities in the Purkinje cell layer. The motor deficits were manifested by impaired rotarod test performance. Further studies employing qRT-PCR analysis demonstrated significant reductions in Hu, MAG-1, and ChAT expression, reflecting reduced populations of neurons, particularly cholinergic, as well as oligodendrocytes. The reduced levels of Hu and ChAT correlate with the conspicuous reductions in granule and Purkinje cell populations, whereas the reductions in MAG-1 expression correspond to hypotrophy or atrophy of central white matter in ethanol-exposed cerebella.

The loss or impaired development of neurons in the granule and Purkinje cell layers, and reductions in MAG-1 expression in ethanol-exposed cerebella were likely due to sustained inhibition of insulin/IGF signaling [17,20,30,31,46]. Insulin and IGF mediate neuronal and oligodendrocyte survival, growth, and metabolism, in addition to neuronal plasticity, myelin maintenance, and cholinergic function [7,8]. Since the binge ethanol exposures were performed within the critical period of robust postnatal cerebellar growth, cerebellar granule cell proliferation, and myelination [47–51] it is conceivable that the toxic effects of ethanol caused a permanent loss or impairment in function of cerebellar neurons and oligodedrocytes. In essence, our findings support the concept that late gestation binge ethanol exposures cause permanent damage to the program of cerebellar development, and thereby produce several of the well-characterized features of FASD [24,28,30].

### Early postnatal binge ethanol exposure causes sustained impairments in cerebellar insulin/IGF signaling

The integrity of upstream signaling through the insulin and IGF-1 receptors was assessed by measuring ligand and receptor gene expression by qRT-PCR analysis, and immunoreactivity corresponding to total and tyrosine phosphorylated insulin and IGF-1 receptors with multiplex ELISAs. The qRT-PCR studies demonstrated similar levels of insulin and IGF-1 polypeptide and receptor gene expression in control and ethanol-exposed cerebella. In contrast, insulin and IGF-1 receptor protein levels were significantly higher in the ethanol-exposed cerebella, marking discordances between mRNA and protein study results. Although tyrosine phosphorylated IGF-1 receptor expression was also increased in ethanol-exposed brains, the relative levels of phosphorylated/total IGR-1 receptor were similar to control. On the other hand, tyrosine phosphorylated insulin receptor levels and the calculated phospho/total insulin receptor levels were significantly reduced by early postnatal binge ethanol exposures. The reduced levels of receptor tyrosine phosphorylation and calculated ratio of phospho/ total insulin receptor immunoreactivity reflect a state of brain insulin resistance. With regard to the IGF-1 receptor, the increased levels of IGF-1R protein and tyrosine phosphorylated IGF-1R suggest that signaling through IGF-1R pathways was increased, perhaps as a compensatory response to insulin resistance.

To better understand the mechanisms of sustained insulin resistance in ethanol-exposed brains, we used competitive saturation assays to measure ligand-receptor binding. Those studies demonstrated that binge ethanol exposure in the early postnatal period leads to significantly impaired insulin as well as IGF-1 receptor binding in adolescent brains. This suggests that both insulin and IGF-1 resistance contributed to the ethanol-associated impairments in cerebellar structure and function in adolescent brains. These results correspond with previous observations in chronic prenatal ethanol exposure models in which we demonstrated that impaired insulin and IGF receptor binding and signal transduction in the perinatal period were mediated by alterations in membrane lipid composition [7,24,28].

With regard to upstream signaling mechanisms, our additional new findings are as follows: 1] ethanol-impaired insulin and IGF-1 signaling persist well beyond the period of exposure and are detectable in young adolescent brains; 2] rather than reducing expression of both the ligands and receptors as occurs in the early postnatal period following chronic prenatal ethanol exposure, postnatal binge ethanol exposures mediate their inhibitory effects on insulin/IGF-1 signaling by impairing ligand-receptor binding, which would likely have resulted in decreased activation of the corresponding receptor tyrosine kinases (at least with respect to the insulin receptor); and 3] although late gestation-equivalent binge ethanol exposures significantly increased IGF-2R and decreased IGF-2 expression, in contrast to the findings in chronic prenatal ethanol exposure models [7,24,28], there were no significant adverse effects of ethanol on IGF-2 receptor binding. It is conceivable that the sustained deficits in brain insulin/IGF-1 signaling might have been compensated for by alternate use of IGF-2 activated networks. However, the effectiveness of this type of response would likely be limited due to reduced levels of IGF-2 polypeptide gene expression in ethanol-exposed brains.

### Effects of early postnatal binge ethanol exposures on IRS expression and signaling in adolescent brains

The stimulatory effects of insulin and IGF-1 are mediated by receptor binding and activation of receptor tyrosine kinases (RTKs) [8,52–58] that phosphorylate IRS proteins. IRSs transmit downstream growth, metabolism, survival, myelin synthesis and myelin homeostasis signals by activating Erk MAPK and PI3K/Akt [16]. Previous studies demonstrated that ethanol impairs insulin and IGF-1 signaling through IRS proteins in various models [23,59], including FASD [24]. The present study shows that early postnatal binge ethanol exposures lead to inhibition of IRS1 and IRS2, but not IRS4 gene expression. Reduced expression of IRS1 and IRS2 compromise the brain’s capacity to transmit signals downstream of the insulin and IGF-1 receptors, and thereby exacerbate states of insulin/IGF-1 resistance, irrespective of the integrity of receptor tyrosine kinase phosphorylation of IRS proteins. However, in contrast to the qRT-PCR analyses, the multiplex ELISA studies demonstrated higher levels of IRS1 protein, and reduced relative levels of pS^312^/total-IRS-1 in the ethanol exposed brains. Since S^312^ phosphorylation of IRS1 inhibits downstream signaling, reduced inhibition corresponds to increased activation. This phenomenon could reflect a compensatory means of supporting downstream signaling vis-à-vis insulin/IGF-1 receptor resistance and thereby account for the fact that cerebellar structure and function are maintained in FASD, albeit at lower levels relative to control.

### Effects of early postnatal binge ethanol exposures on Akt pathway signaling in the cerebellum

Ethanol has profound inhibitory effects on insulin/IGF signaling through PI3K/Akt in immature neurons and the developing brains [27,30,34,60]. Ethanol mediates these effects by: 1] inhibiting IRS-associated PI3K activity, and subsequent phosphorylation and activation of Akt and phosphorylation/inhibition of GSK-3β [7,19,23– 27]; and 2] increasing the activity of phosphatases that negatively regulate receptor tyrosine kinases, e.g. PTP-1b and PI3K (PTEN) [24– 26]. Akt promotes cell survival, cell migration, energy metabolism, and neuronal plasticity, and it inhibits GSK-3β activity. Consequently, ethanol inhibition of PI3K-Akt leads to increased GSK-3β-mediated oxidative stress, DNA damage, mitochondrial dysfunction, apoptosis, and disordered neuronal migration [16,27,60,61]. The reduced relative levels of S^473^ phosphorylation of Akt and S^9^ phosphorylation of GSK-3β in ethanol-exposed young adolescent cerebella indicates that the inhibitory effects of ethanol on signaling downstream of the insulin receptor are sustained beyond the period of exposure and likely mediated by persistent brain insulin resistance as discussed earlier [7].

In conclusion, early postnatal binge ethanol exposures cause long-term deficits in motor function associated with structural abnormalities in the cerebellum, including hypocellularity and hypofoliation. These adverse effects were likely due to sustained inhibition of signaling through insulin/IGF-1 pathways, and downstream through IRS and PI3K/Akt. Future studies will determine the degree to which restoration of insulin/IGF signaling with insulin sensitizer agents abrogates structural and functional abnormalities in the cerebellum.

## Figures and Tables

**Table 1:** Effects of Early Postnatal Binge Ethanol Exposures on Insulin/IGF Receptor Binding in Young Adolescent Cerebella.

	Control Insulin	Ethanol Insulin	Control IGF1	Ethanol IGF1 IGF1	Control IGF2	Ethanol IGF2
**Best-fit values**						
Bmax	4.92 ± 0.82	3.01 ± 0.41*	11.56 ±0.99	8.6 ± 0.87**	25.3 ± 3.25	25.06 ± 2.19
Kd	4.06 ± 1.01	2.33 ± 0.55	1.17 ± 0.26	0.99 ± 0.28	1.89 ± 0.51	2.56 ± 0.43
Bmax [95% CIL]	3.29–6.63	2.19–3.83	9.54–13.58	6.85–10.39	18.73–31.88	20.63–29.48
Kd [95% CIL]	2.01–6.11	1.22–3.45	0.64–1.70	0.42–1.55	0.85–2.93	1.72–3.47
**Goodness of fit**						
R-square	0.931	0.903	0.882	0.820	0.851	0.940

Bmax=Top-level binding; Kd=binding affinity constant; CIL= confidence interval limits

* Inter-group statistical comparisons for insulin receptor binding: Bmax, P=0.017; Kd, P=0.09

** Inter-group comparisons for IGF1 receptor binding: Bmax, P=0.022; Kd, P=0.60
